# A Review of the Effect of Diet on Cardiovascular Calcification

**DOI:** 10.3390/ijms16048861

**Published:** 2015-04-21

**Authors:** Rachel Nicoll, John McLaren Howard, Michael Y. Henein

**Affiliations:** 1 Department of Public Health and Clinical Medicine and Heart Centre, Umea University, Umea SE-901 87, Sweden; E-Mail: michael.henein@umu.se; 2 Acumen Lab, Tiverton, Devon EX16 6BL, UK; E-Mail: acumenlab@hotmail.co.uk

**Keywords:** cardiovascular calcification, coronary calcification, diet, vitamins, minerals, antioxidants, homocysteine, transfats, ω-3 fats

## Abstract

Cardiovascular (CV) calcification is known as sub-clinical atherosclerosis and is recognised as a predictor of CV events and mortality. As yet there is no treatment for CV calcification and conventional CV risk factors are not consistently correlated, leaving clinicians uncertain as to optimum management for these patients. For this reason, a review of studies investigating diet and serum levels of macro- and micronutrients was carried out. Although there were few human studies of macronutrients, nevertheless transfats and simple sugars should be avoided, while long chain ω-3 fats from oily fish may be protective. Among the micronutrients, an intake of 800 μg/day calcium was beneficial in those without renal disease or hyperparathyroidism, while inorganic phosphorus from food preservatives and colas may induce calcification. A high intake of magnesium (≥380 mg/day) and phylloquinone (500 μg/day) proved protective, as did a serum 25(OH)D concentration of ≥75 nmol/L. Although oxidative damage appears to be a cause of CV calcification, the antioxidant vitamins proved to be largely ineffective, while supplementation of α-tocopherol may induce calcification. Nevertheless other antioxidant compounds (epigallocatechin gallate from green tea and resveratrol from red wine) were protective. Finally, a homocysteine concentration >12 µmol/L was predictive of CV calcification, although a plasma folate concentration of >39.4 nmol/L could both lower homocysteine and protect against calcification. In terms of a dietary programme, these recommendations indicate avoiding sugar and the transfats and preservatives found in processed foods and drinks and adopting a diet high in oily fish and vegetables. The micronutrients magnesium and vitamin K may be worthy of further investigation as a treatment option for CV calcification.

## 1. Introduction

Cardiovascular (CV) calcification is a systemic disease [1] and is an independent predictor of CV events and all-cause mortality in both CV and renal patients [2,3,4,5], while coronary artery calcification (CAC) scoring provides improved predictive ability over conventional risk factor scoring alone [6,7]. Although CAC may be present both with and without severe flow-limiting lesions, in view of its common occurrence as calcification of the atheroma cap, it has become known as a form of subclinical atherosclerosis and is widely used as a marker for coronary artery disease (CAD) [5]. The initiation of CV calcification resembles the process of osteogenesis, involving the same cells, proteins and cytokines. Studies have shown osteoblast- and osteoclast-like cells and bone matrix proteins in the arterial wall [8], hydroxyapatite (calcium phosphate) deposition and, in cases of more severe calcification, fully formed bone is seen in arteries and valves [5,9,10]. It remains unclear as to why CV calcification forms in the first place but its pathophysiology is complex, involving not only physicochemical factors but also biological actions in smooth muscle [11].

At present there is no specific treatment for arterial calcification; medications such as statins, vasodilators and other therapy for atherosclerosis and calcific aortic stenosis have negligible effect, although they are beneficial in lowering low density lipoprotein (LDL), a key risk factor for CAD, preventing against development of flow-limiting lesions and reducing inflammation, an important cause of atherosclerosis [12,13]. Similarly, the conventional CV risk factors of dyslipidaemia, hypertension, diabetes, family history, obesity and smoking are not consistent predictors of CAC [14,15]. Consequently, a review of dietary factors was carried out in an attempt to determine whether there were any significant associations with CV calcification. PubMed was searched for articles on CV calcification and diet or nutrition, with specific searches on vitamins, minerals and other dietary components. The majority of diet studies assessed intake by validated questionnaire, although a few used 24 h recall. Since there were few studies of calcification and nutrition, this review was extended to include serum and plasma concentrations of the dietary macro- and micronutrients.

## 2. Fatty Acids

The few studies of total and saturated fat intake and CV calcification presence or extent show mixed results, although intake of transfats was positively associated [16,17,18]. There have been no studies of MUFAs but a small study of Korean haemodialysis patients found no association of intake of ω6 PUFAs and calcification of the extremities or the abdominal aorta [18]. Among the studies of fish and fish oils, a large US study found no association of intake of long chain ω3 PUFAs and non-fried fish with CAC presence [19] and in two studies comparing Japanese and Caucasian men, one showed no association with CAC presence after adjustment for CVD risk factors [20] but the second found that Japanese men had a significantly higher level of serum long chain ω3 PUFAs and a lower incidence of CAC compared to Caucasians, even after adjusting for conventional CV risk factors [21]. In Japanese acute myocardial infarction (MI) patients, the log of a serum long chain ω3 PUFA predicted the CAC score [22]. A large Dutch prospective study found no difference among those with fish intake >19 g/day compared to zero and the CAC score, although the majority of the fish consumed was cod which does not contain high long chain ω3 PUFAs [23].

There are no intervention studies of fatty acids and CV calcification but in animals, dietary cholesterol is regularly used to induce calcification in models of hypercholesterolaemia [24]. A diet rich in linoleic acid (LA), an ω6 PUFA, decreased heart valve and gastric and renal artery calcification in animals [25], while fish oil and another ω6 PUFA, γ-linolenic acid (GLA), significantly reduced renal calcification [26]. Fish oil could also limit induced abdominal aorta calcification (AAC) [27] and, when given with antioxidant lipoic acid, prevented aortic and renal calcification [28]. In *in vitro* studies, transfats increased the incorporation of calcium into human arterial endothelial cells [29], while ω3 fats inhibited the mineralization of vascular cells and activity of alkaline phosphatase (ALP), a marker of bone formation [30].

## 3. Carbohydrates

Carbohydrates comprise starches (complex carbohydrates) and sugars (simple carbohydrates). There are only a few studies measuring calcification but these are at variance, with a prospective study of premenopausal women showing that carbohydrate intake was inversely associated with coronary, but not aortic calcification, five years after menopause [16], while a large cross-sectional study showed that whole grain intake was not associated with CAC [31]. Although there are no studies considering dietary intake of sugars, diabetes mellitus, metabolic syndrome, insulin resistance and high blood glucose, HbA1C and triglycerides are known risk factors for the presence of aortic [32], carotid [33] and coronary artery calcification [34,35] and may also correlate with the aortic calcification [36] or CAC score [37]. The relationship between CAC presence and blood glucose levels was found particularly among men [38], with low insulin levels, as well as hyperinsulinaemia, independently predicting CAC presence [39]. Similarly, a high glucose medium enhances calcification of human vascular smooth muscle cells (VSMCs) and increases expression of markers of bone formation [40]. In animals, starch or sugar can result in increased incidence of CV calcification [41]; only galactooligosaccharides (prebiotics) can reduce CV calcification [42].

## 4. Protein

There have been no human studies investigating protein intake and CV calcification and in animals they focus almost exclusively on nephrocalcinosis, with the majority showing that increased dietary protein reduced renal calcification in female rats with or without chronic kidney disease (CKD) [43,44]. Likewise, a low protein intake induced more severe renal calcification, with possible kidney damage [45].

## 5. Minerals

This review covers only calcium, phosphorus and magnesium, as there were no human or animal studies investigating the effect of other minerals on CV calcification.

### 5.1. Calcium

Calcium fulfils vital roles in the body, particularly with respect to cell signalling functions; for this reason it is critical that serum calcium be maintained in a very narrow range [46]. Possibly for this reason, intake often bears little relationship to serum calcium levels [47] and observational studies generally show little association of dietary or supplemental intake, with CAC or abdominal aortic calcification (AAC) incidence or extent in older adults [48,49,50,51,52], although a large study showed that calcium intake was significantly higher in postmenopausal women without AAC at baseline and after five years [47]. In animals, low calcium intake induces higher nephrocalcinosis and aortic calcium content, while high calcium intake is not generally associated with CV calcification in health [53,54]. However, in rats with CKD and secondary hyperparathyroidism, calcium supplementation increased arterial calcification [55]. Studies of serum calcium are mixed but concentrations are largely unaffected by intake [47,56,57,58].

### 5.2. Phosphorus

As with calcium, phosphate is important for cell signalling and energy storage in the form of ATP, requiring strict control over blood concentrations [59]. In the few studies of the effect of dietary phosphorus there is no association with CAC in older Koreans [51], although animal studies showed a positive association between phosphorus intake and aortic and renal calcification [60]. Two large studies by Linefsky *et al.* found a significant association between mean serum phosphate levels (>1.292 *vs.* ≤0.969 mmol/L) and aortic valve calcification (AVC) and mitral annulus calcification (MAC) but not AAC presence, though after a mean of 2.4 years showed no association with extent, progression or new development [57,61]. These studies demonstrate that even a serum phosphate value within the reference range (0.8–1.4 mmol/L) is associated with valve calcification [62]. Studies of older adults and CKD patients also found a significant association between higher serum phosphate and presence and extent of calcification [51,56,58,63,64], with each 0.0323 mmol/L rise in serum phosphorus being associated with 6.1% higher odds of having CAC [56]. The association with AAC may be particularly relevant in females [47]. In CKD, arterial calcification can be suppressed by reducing serum phosphate levels [65]. VSMCs cultured in normal levels of phosphorus do not calcify [66] but a high phosphorus medium, particularly inorganic phosphorus, can induce calcification [56,60,66,67,68].

### 5.3. Magnesium

Magnesium is an essential mineral, acting as cofactor in more than 300 enzymatic reactions. It is a natural calcium channel blocker and plays an important role in CV, neurological and metabolic functions [69]. The principal intake study found that CV calcification was lowest in the highest quartile, with intake ranging from 384 to 669 mg/day [70]. The only studies of blood concentrations involve dialysis patients and show a clear association between lower serum magnesium (1.1056 *vs.* 1.241 mmol/L) and incidence of peripheral artery calcification [71,72] and MAC [73]. In animals, a low magnesium diet increased cardiac mineral deposition [74,75], the effect being exacerbated when low magnesium intake was combined with high phosphorus [75]. There are no trials of magnesium supplementation on CV calcification in humans, but in animals supplementation dose-dependently lowered myocardial, carotid and aortic calcium content [76,77]. Similarly, *in vitro* studies showed that increasing magnesium concentration reduced calcification in VSMCs [68,74].

## 6. Vitamin D

Vitamin D is a steroid hormone that has multiple roles in the body, including the CV system [78], but of principal importance is its endocrine function, which includes maintenance of serum calcium within a narrow range [79]. This can often operate to the detriment of other functions [80], which may account for the lack of clear results in vitamin D studies. There are no human intake studies with respect to CV calcification, probably because dietary vitamin D provides only a relatively small contribution to serum 25(OH)D. Animal studies, however, show that high vitamin D intake can induce CV calcification [81,82] but they also show that a vitamin D-deficient diet can induce an increase in calcified lesions [83,84,85], indicating that both excess and deficiency are detrimental.

Several epidemiological studies measuring serum 25(OH)D demonstrate no association with presence or extent of CAC or MAC [86,87], although after three years low serum 25(OH)D was associated with new CAC development, but not CAC progression [88]. There is often an inverse association with calcification presence in those with previously diagnosed disease, such as CKD [89,90], type 1 diabetes [91] or dilated cardiomyopathy [92], where a serum 25(OH)D concentration of ≥75 nmol/L is protective. Likewise with respect to serum 1,25(OH)2D, some studies show no association with CAC extent or progression [87,93], although in subjects at risk for CHD, serum 1,25(OH)2D was inversely correlated with the extent of calcification [94]. There have been few intervention studies of vitamin D alone but in CKD patients the incidence of aortic calcification was significantly lower in treated patients [95]. Trials of vitamin D supplementation combined with calcium showed that up to 1000 g/day calcium plus 400 IU/day vitamin D3 did not affect CAC scores or incidence of MI, CHD mortality or stroke in postmenopausal women [96,97]; this lack of result may be because the vitamin D dose was low.

## 7. Vitamin K

Vitamin K, formerly thought to be merely a coagulation inducer, is now exciting considerable interest over its effect on CV calcification. Fat-soluble vitamin K occurs naturally in two forms: phylloquinone (vitamin K1), which is the more commonly ingested, and 14 forms of menaquinone (vitamin K2), of which MK4 and MK7 are the most studied. Both forms of vitamin K can function as enzyme cofactors in the γ-carboxylation of glutamic acid residues to produce the calcium-binding γ-carboxyglutamate (Gla) proteins. The principal vitamin K-dependent protein involved in CV calcification is matrix Gla protein (MGP), produced in VSMCs. Once MGP is γ-carboxylated, the resultant Gla residues give it a calcium ion-binding property but if the protein is undercarboxylated, calcium binding can be severely attenuated [98]. This calcium binding property may determine whether hydroxyapatite ends up in bone (full carboxylation) or arteries (undercarboxylation). Many patients with CV calcification, however, may also be on warfarin, a vitamin K antagonist, which blocks the γ-carboxylation of MGP [99]; these patients have significantly increased calcification presence and extent [100,101,102].

Studies of phylloquinone intake generally show no association with the CAC score, CAC progression or AAC presence [103,104,105,106,107], although menaquinones may have more effect in arteries. In postmenopausal women, a higher intake of MK4 (48.5 *vs.* 18 μg/day) was associated with a lower CAC score [103] and was inversely associated with severe AAC (28.8 *vs.* 25.6 μg/day) [104] but not in all studies [107]. Similarly, there was no correlation between serum phylloquinone concentrations and extreme CAC progression [105] or presence of AVC, MAC or TAC in postmenopausal women after 8.5 years [108], although one study found that serum phylloquinone was positively associated with increased CAC presence [109]. By contrast, serum MK4 deficiency predicted aortic calcification, while MK7 deficiency predicted iliac calcification in CKD patients [106]. In the only human trial investigating CV calcification, 500 μg/day phylloquinone supplemented for three years in older adults decreased plasma ucMGP, resulting in significantly less CAC progression [110]. Similarly, animal and *in vitro* studies also show that phylloquinone and MK4 reduce CV and renal calcification [111,112], with MK4 proving the more effective [113]. Vitamin K may work indirectly to prevent calcification through γ-carboxylation of its dependent proteins, with most studies showing an association [107,114,115,116].

## 8. Antioxidants

CV calcification is particularly prevalent in patients with diabetes and CKD, largely due to increased reactive oxygen species (ROS) and lipid oxidation products such as malondialdehyde [82,117]. Excess ROS induces an imbalance between cellular and tissue pro-oxidants and endogenous antioxidant enzymes, such as superoxide dismutase (SOD), catalase (CAT) and glutathione peroxidase (GPx), which are in part dependent on intake of dietary nutrients [118]. Among the relatively few studies to have assessed the impact of oxidative stress on CV calcification, Ahmadi *et al.* showed a positive correlation between CAC progression and serum malondialdehyde [119], while Watanabe *et al.* showed that type 2 diabetics with aortic arch calcification had significantly higher levels of oxygen metabolites, which were more predictive of calcification than markers of inflammation [120]. Similarly, in induced animal aortic valve calcification several ROS species were upregulated [121], while oxidative stress induced aortic calcification and upregulation of osteoblastic proteins in VSMCs [122,123]. The case for a high antioxidant status as an inhibitor of calcification is not so clear, however. Valabhji *et al.* found that the inverse association between total antioxidant status and CAC became non-significant after adjustment for age or diabetes duration [124].

### 8.1. Vitamin A and Carotenoids

Vitamin A is a fat-soluble vitamin, critical for vision, growth regulation and cell differentiation and comprises retinol and carotenoids [125]. Among the few human studies of vitamin A, none has shown a correlation between dietary or serum vitamin A and CAC score [126], although a study of menopausal women found a U-shaped association between serum retinol-binding protein (RBP) 4 and CAC prevalence [127]. Animal studies, however, showed that retinyl palmitate and retinoic acid treatment induce valve calcification, with increased expression of osteogenic genes [128]. There are no human carotenoid intake investigations and among the few serum studies there were no significant associations between incidence of calcified aortic plaque and serum lycopene, α-carotene, β-carotene, lutein or zeaxanthin levels, even in current or former smokers who are known to have increased oxidative stress [129,130]. Nevertheless, in the second and third quartiles of serum β-cryptoxanthin, an increased risk of aortic calcification was observed [130], indicating an inverse U-shaped association.

### 8.2. Vitamin C

Vitamin C (ascorbic acid) is an essential cofactor in enzymatic reactions [131]. In observational studies intake of vitamin C was not associated with the CAC score in asymptomatic subjects [126], although intervention studies using a combination of a statin, ascorbic acid and α-tocopherol had no effect on the CAC score [132]. Nevertheless, ascorbic acid plus α-tocopherol reduced the calcification found in pseudoxanthoma elasticum [133] and may lower induced calcification in VSMCs [122,134].

### 8.3. Vitamin E

Vitamin E collectively refers to the ten fat-soluble, chemically distinct isoforms: α, β, γ, δ and ε tocopherols and tocotrienols [135]. The commercial availability of vitamin E is mostly in the form of α-tocopherol, which is the most abundant form of vitamin E in human tissues and serum [136]. Two cross-sectional studies showed that in healthy middle-aged subjects and older haemodialysis patients, there was no correlation between the CAC score [126] or peripheral arterial calcification score [18] and dietary vitamin E but when supplemental intake (likely α-tocopherol) was also assessed, those supplementing had a significantly higher risk of CAC than those not supplementing (105.5 *vs.* 76.4 mg/day) [126]. A large intervention study of adults with CAC scores ≥80th percentile for age and gender, showed that a combination of a statin, vitamin C and 1000 IU/day α-tocopherol for a mean of 4.3 years had no effect on CAC progression [132].

### 8.4. Flavonoids and Polyphenols

Flavonoids and polyphenols are known to have powerful antioxidant effects, particularly those found in tea and coffee, such as epigallocatechin gallate (EGCG), rutin and quercetin, and red wine (resveratrol), while the barley and hops used to make beer are also rich polyphenol sources. Two large studies of older adults investigated coffee intake but showed mixed results, with one finding that coffee drinking was independently correlated with aortic calcification presence [137], while another showed that >3 daily cups of coffee significantly reduced incidence of a CAC score of >400 [138]. EGCG reduced renal calcification in rats [139], while quercetin attenuated VSMC and porcine heart valve calcification [140,141] and reduced CKD rat aorta calcification, with catechin and rutin being partially effective [142]. Human studies of alcohol intake show mixed results, which may be due to a J-shaped association and different effects according to alcohol type [143,144]. Few studies stratify by type of alcohol but nevertheless, resveratrol supplementation reduced aortic calcification and atherosclerotic lesions in animals [145]. Similarly, *in vitro* studies found that the polyphenols in wine and beer were able to concentration-dependently inhibit VSMC alkaline phosphatase activity [140].

### 8.5. Other Antioxidants

α-Lipoic acid (ALA) is made in the human body and is essential for aerobic metabolism and as a cofactor in several enzymatic reactions. There are no human studies of the effect of ALA on CV calcification but in mice with calcification from heart tissue injury, ALA lowered tissue damage and reduced calcification [146], while in rabbits it reduced aortic valve and medial calcification [121,147]. *N*-acetylcysteine (NAC) functions principally as a mucolytic agent and helps to increase endogenous glutathione levels. Again, there are no human studies of NAC and CV calcification but in mice with oxidative stress-induced aortic calcification, NAC slowed its progression [148], while in hypertensive uraemic rats, NAC suppressed markers of cellular senescence associated with arterial calcification [149]. *In vitro* studies showed that NAC could inhibit osteoblast apoptosis and calcification VSMCs [150].

## 9. B Vitamins and Homocysteine

The role of the water-soluble B vitamins is principally energy production, cell metabolism and nerve function. In addition, vitamins B12, B6 and folic acid have a function in lowering homocysteine, a sulphur-containing amino acid and excitatory neurotransmitter produced naturally in the body [151]. Meta-analyses of B vitamin trials consistently show that ≥0.5 mg/day folic acid dose-dependently reduced homocysteine, with the addition of 0.4 mg/day vitamin B12 producing a further 7% reduction [152,153,154,155]. A 2007 systematic review revealed a significant positive association between elevated levels of homocysteine and presence of coronary artery calcification (CAC) in most studies [156]; these findings have generally been confirmed in arteries in later studies [157,158,159] but not in the aortic valve [160]. The carotid calcification score increased across quartiles of plasma homocysteine (<9.4 *vs.* >13.4 μmol/L) [161], while CAC progression was twice as rapid in individuals with plasma homocysteine concentrations of ≥12 μmol/L [162]. The association in CKD patients is weaker [163,164,165].

Despite the association of high homocysteine with CV calcification and the evidence that B vitamins can lower homocysteine, there have been no observational studies investigating intake of B vitamins and incidence of CV calcification and only one study investigating serum levels. Here, the carotid calcification score decreased across quartiles of plasma folate concentrations (>39.4 *vs.* <23 nmol/L) in patients with vascular disease or diabetes but there was no association with vitamin B6 or B12 concentrations [161]. In the only clinical trial of B vitamins alone, subjects with baseline plasma homocysteine levels of >8.5 nmol/L were given 5 mg/day folic acid, 0.4 mg/day vitamin B12 and 50 mg/day vitamin B6 for three years but there was no significant effect on progression of coronary or aortic calcification [166]; this may be because a mean baseline homocysteine level of 9.5 nmol/L is possibly too low for it to be responsible for inducing subclinical atherosclerosis. Nevertheless, two trials showed that a combination of 0.1 mg/day vitamin B12, 0.3 mg/day folic acid, 12.5 mg/day vitamin B6, aged garlic extract and l-arginine for 12 months produced significantly lower homocysteine and CAC progression [167,168].

## 10. Discussion

There are few human studies of macronutrients, but in general transfats and disordered blood glucose and insulin should be avoided, while long chain ω3 PUFAs may be protective against CV calcification. Among the micronutrients, most human studies of calcium and phosphorus intake show little association, although a higher calcium intake may be protective. However, higher serum phosphate levels, even within the reference range, were associated with calcification. In contrast, the protective effect of a higher magnesium intake (≥380 mg/day) is clearer and animal studies show promise for the use of magnesium to lower CV calcification. Epidemiological studies of vitamin D indicate that a higher serum level (≥75 nmol/L) may be protective, while supplementation lowered aortic calcification in CKD patients, with the association being clearer in patients with diagnosed disease. It is difficult to assess the effects of vitamin D alone, since its predominant function is to maintain serum calcium homeostasis and it will do this to the detriment of arteries if necessary; baseline serum calcium is seldom measured in these studies.

Although a trial of 500 μg/day phylloquinone significantly reduced CAC progression, observational and animal studies suggest that menaquinone has greater promise. Oxidative stress, common in diabetes and CKD, correlated with calcification incidence and progression, although the association with dietary antioxidants was less clear. In human studies, there was little correlation between CV calcification and dietary vitamin A, vitamin C or carotenoids, but supplementary vitamin E (probably as α-tocopherol) was associated with higher risk of CAC. ALA, NAC, EGCG, resveratrol and other isomers of vitamin E show promise for protecting against calcification in animal studies. Similarly, there was a significant positive association between homocysteine levels (threshold around 12 μmol/L) and arterial calcification, vitamins B6, B12 and folate have been shown to lower homocysteine levels and serum folate levels were inversely associated with calcification extent. Nevertheless, in the only trial of B vitamins alone, supplementation had no effect but this may be because in these patients homocysteine was unlikely to be the cause of calcification. Two trials of B vitamins combined with garlic and l-arginine significantly reduced CAC progression. A summary is shown in Table 1.

**Table 1:** Dietary and serum recommendations from human studies of cardiovascular calcification.

Macro/Micro-Nutrient	Recommended Intake	Recommended Serum Level	Source
Trans fats; [18,29]	Avoid	N/A	Hydrogenated oils, spreads, processed foods, heated PUFAs
Long chain ω3 fats; [21,22,26,27,28,30]	High intake	N/A	Oily fish
Sugars; [31,32,33,34,35,36,37,38,39,40,41]	Avoid	Low glucose, mid-range insulin	Sugar, sweets, drinks, fruit juice
Calcium; [47,53,54,55]	800 mg/day beneficial provided no CKD or hyper-parathyroidism	N/A	Dairy products, fish, legumes, grains, vegetables
Inorganic phosphorus; [57,61,62]	Avoid	N/A	Preservatives, colas
Magnesium; [70,71,72,73]	≥380 mg/day	N/A	Vegetables, nuts, seeds, fruits, grains, legumes, fish, dairy foods
Vitamin D; [88,89,90,91,92,94,95]	N/A	≥75 nmol/L	Sunlight, egg yolk, offal, oily fish, shellfish, fortified foods
Vitamin K (phylloquinone); [103,104,110]	Beneficial at 500 μg/day	N/A	Vegetables, supplements
Vitamin E (α-tocopherol); [126]	Avoid	N/A	Supplements
Epigallocatechin gallate (EGCG); [139]	Beneficial	N/A	Green tea
Resveratrol; [140,145]	Beneficial	N/A	Red wine, red grape juice
Homocysteine; [156,157,158,159,161,162]	N/A	≤12 μmol/L	N/A
Folate; [161]	Beneficial	>39.4 nmol/L	Green leafy vegetables, wholegrains, nuts, fortified cereals

N/A indicates that there is no recommended intake or serum level in the literature.

There are insufficient studies of intake and CV calcification to draw firm parallels with the dietary risk factors for coronary artery disease (CAD), although in general the advice for prevention of calcification would also help prevent CAD. Nevertheless, a consistent theme throughout the nutrient intake studies is oxidative damage as a cause of CV calcification and CAD. Elevated lipid oxidation products and oxidised LDL are thought to be responsible for both atherosclerosis and mineralisation of vascular cells [169,170,171,172]. Advanced glycation end products (AGEs), known to be elevated in diabetes, generate ROS when in contact with their receptor (RAGE) [173] and induce VSMC calcification [174], which could be inhibited by antioxidants [175,176]. Furthermore, homocysteine is a pro-oxidant, whose effect could be inhibited by antioxidants [177,178,179], while other compounds with antioxidant effect include vitamin K-hydroquinone, which can suppress lipid peroxidation [180], and MK4, which can inhibit oxidant-induced inflammation [181].

## 11. Conclusions

Although there are insufficient studies of intake or serum levels of nutrients to draw firm conclusions, in general transfats, disordered blood glucose and insulin, higher serum phosphate levels, oxidative stress and α-tocopherol supplementation should be avoided, while intake of long chain ω3 PUFAs, calcium, magnesium, vitamins B, D and K and antioxidants should be increased. In practice this amounts to avoiding sugar and processed foods and increasing oily fish, fruits and vegetables. Although animal studies have shown that many of the micronutrients discussed here may be effective in lowering or preventing CV calcification, there have been remarkably few clinical trials. In particular, it would be worth testing the effects of long chain ω3 PUFAs, magnesium and vitamin K.
